# Death Receptors DR4 and DR5 Undergo Spontaneous and Ligand-Mediated Endocytosis and Recycling Regardless of the Sensitivity of Cancer Cells to TRAIL

**DOI:** 10.3389/fcell.2021.733688

**Published:** 2021-09-30

**Authors:** Artem A. Artykov, Anne V. Yagolovich, Dmitry A. Dolgikh, Mikhail P. Kirpichnikov, Daria B. Trushina, Marine E. Gasparian

**Affiliations:** ^1^Department of Bioengineering, Institute of Bioorganic Chemistry (RAS), Moscow, Russia; ^2^Faculty of Biology, Lomonosov Moscow State University, Moscow, Russia; ^3^Department of X-Ray and Synchrotron Research, A.V. Shubnikov Institute of Crystallography of Federal Scientific Research Centre “Crystallography and Photonics” of Russian Academy of Sciences, Moscow, Russia

**Keywords:** DR5, DR4, receptor endocytosis, receptor recycling, membrane traffic, brefeldin A

## Abstract

Tumor necrosis factor-associated ligand inducing apoptosis (TRAIL) induces apoptosis through the death receptors (DRs) 4 and 5 expressed on the cell surface. Upon ligand stimulation, death receptors are rapidly internalized through clathrin-dependent and -independent mechanisms. However, there have been conflicting data on the role of death receptor endocytosis in apoptotic TRAIL signaling and possible cell type-specific differences in TRAIL signaling have been proposed. Here we have compared the kinetics of TRAIL-mediated internalization and subsequent recycling of DR4 and DR5 in resistant (HT-29 and A549) and sensitive (HCT116 and Jurkat) tumor cell lines of various origin. TRAIL stimulated the internalization of both receptors in a concentration-dependent manner with similar kinetics in sensitive and resistant cell lines without affecting the steady-state expression of DR4 and DR5 in cell lysates. Using the receptor-selective TRAIL variant DR5-B, we have shown that DR5 is internalized independently of DR4 receptor. After internalization and elimination of TRAIL from culture medium, the receptors slowly return to the plasma membrane. Within 4 h in resistant or 6 h in sensitive cells, the surface expression of receptors was completely restored. Recovery of receptors occurred both from newly synthesized molecules or from trans-Golgi network, as cycloheximide and brefeldin A inhibited this process. These agents also suppressed the expression of cell surface receptors in a time- and concentration-dependent manner, indicating that DRs undergo constitutive endocytosis. Inhibition of receptor endocytosis by sucrose led to sensitization of resistant cells to TRAIL and to an increase in its cytotoxic activity against sensitive cells. Our results confirm the universal nature of TRAIL-induced death receptor endocytosis, thus cell sensitivity to TRAIL can be associated with post-endocytic events.

## Introduction

Cell surface receptor uptake and subsequent intracellular sorting for degradation or recycling regulates the specificity of downstream signaling. Some receptors are internalized continuously whereas others remain on the surface until a ligand is bound. In cancer cells the endocytic trafficking of signaling receptors such as receptor tyrosine kinases (RTKs), G protein–coupled receptors (GPCRs), and cytokine receptors is altered, affecting signaling pathways to enhance tumorigenesis and metastasis (Mellman and Yarden, 2013; Cendrowski et al., 2016; Schmid, 2017).

Ligand-mediated receptor internalization plays an important role in tumor necrosis factor (TNF) family member receptor signaling. Internalization of TNF-R1 and CD95 receptors is required for TNF- and FasL-mediated apoptosis signaling (Schütze and Schneider-Brachert, 2009; Schneider-Brachert et al., 2013). Studies have also been conducted to elucidate the role of death receptor internalization in binding to TNF associated ligand inducing apoptosis (TRAIL). TRAIL induce apoptosis in cancer cells by activating death receptors DR4 and DR5 (LeBlanc and Ashkenazi, 2003). After stimulation with ligands, DR4 and DR5 rapidly internalize, and the role of this process in the regulation of TRAIL-mediated apoptosis is unclear. In some studies, inhibition of endocytosis by specific molecules or inactivation of dynamin increased TRAIL-mediated apoptosis (Kohlhaas et al., 2007; Zhang and Zhang, 2008; Zhang et al., 2009). Recently it was demonstrated that TRAIL selectively activated dynamin-1 to self-regulate death receptors endocytosis, attenuate apoptotic signaling and increase cell survival (Reis et al., 2017). In addition, clathrin-independent mechanisms were also suggested to participate in TRAIL death receptor internalization (Kohlhaas et al., 2007). Blocking caveola-mediated DR4 internalization by filipin III enhanced TRAIL-induced apoptosis (Zhao et al., 2009).

In contrast to the aforementioned studies, TRAIL-induced internalization of death receptors was proved important for apoptosis signaling. TRAIL-induced DR5 internalization is necessary for permeabilization of lysosomal membranes and apoptosis in malignant liver cells (Akazawa et al., 2009). Dominant-negative dynamin mutant and Rab7 silencing inhibit apoptotic signaling in human hepatocellular carcinoma cell lines Huh-7 and HNU 499, cholangiocarcinoma cell lines Mz-ChA-1 and HuCCT-1, but not in human cervical cancer cell line HeLa. Increased surface expression of endogenous lectin galectin-3 in metastatic colon adenocarcinoma LiM6-TR cells prevented the endocytosis of TRAIL receptors. Reduction of galectin-3 expression restored endocytosis of TRAIL receptors and TRAIL-dependent apoptosis (Mazurek et al., 2012). Thus, the role of internalization of TRAIL death receptors for signaling apoptosis is cell type-dependent. While DISC (death inducing signaling complex) formation and activation of caspase 8 at the plasma membrane are sufficient to induce apoptosis in type I cells, the induction of TRAIL-mediated apoptosis in type II cells may strongly depend on receptor internalization (Kohlhaas et al., 2007; Akazawa et al., 2009).

In addition to TRAIL-induced internalization, death receptors can also undergo constitutive endocytosis as a part of desensitizing mechanism. DR4 and DR5 constitutive endocytosis in breast cancer cell lines decreased their surface expression regardless of mRNA and total protein levels leading to TRAIL resistance (Zhang and Zhang, 2008).

A growing body of evidence indicates that the subcellular localization and the regulation of membrane transport of death receptors play an important role in determining apoptotic and non-apoptotic TRAIL signaling (Bertsch et al., 2014). In addition to its canonical location in the plasma membrane, TRAIL death receptors have been identified in the endosomes, lysosomes, autophagosomes, in the cytosolic compartment as well as in the nucleus (Zhang et al., 2000; Akazawa et al., 2009; Leithner et al., 2009; Di et al., 2013; Haselmann et al., 2014). It was also demonstrated that colon carcinoma cells can secrete extracellular vesicles coated with DR5 receptor, and competitive binding of TRAIL to DR5 on target cells and DR5 on vesicles leads to a decrease in apoptosis signaling (Setroikromo et al., 2020). The nuclear DR5 inhibits maturation of the microRNA let-7 in pancreatic cancer cell lines and increases their proliferation (Haselmann et al., 2014). Importin β1-mediated nuclear localization of DR5 limits TRAIL-induced death of tumor cells (Kojima et al., 2011). Later, the authors demonstrated that inhibition of importin β1 enhances the anticancer effect of an anti-DR5 agonist antibody in TRAIL-resistant tumor cells (Kojima et al., 2020). Recent studies demonstrated that death receptors DR4 and DR5 are constitutively localized to chromatin from the plasma membrane via clathrin-dependent endocytosis, and this process is greatly enhanced by TRAIL-mediated receptor endocytosis (Mert et al., 2019).

In this study, we showed that the death receptors DR4 and DR5 undergo constitutive and ligand-stimulated endocytosis with similar kinetics in TRAIL-sensitive and TRAIL-resistant tumor cell lines. Using the receptor-selective TRAIL variant DR5-B, we proved that death receptors can be internalize independently of each other. After internalization, the receptors slowly returned to the plasma membrane when TRAIL was washed out from culture medium. Within 6 h the surface expression of receptors was completely restored, regardless of the sensitivity of the tumor cells to TRAIL. The levels of receptors were restored through a combination of newly synthesized protein and recycling from endocytic compartments. Inhibition of receptor endocytosis by sucrose sensitized resistant cells to TRAIL and increased its cytotoxic activity against sensitive cells.

## Materials and Methods

### Cell Lines and Culture Conditions

Human colorectal adenocarcinoma cell lines HT-29 and HCT116, Jurkat T-lymphoblastic leukemia cells, A549 human lung adenocarcinoma cell line were purchased from the Research Institute of Cytology, Russian Academy of Sciences (St. Petersburg, Russia). Nutrient medium DMEM supplemented with 10% fetal calf serum was used to cultivate A549 and HCT116 cells and RPMI1640 medium supplemented with 10% fetal calf serum for HT-29 and Jurkat cells. All cells were cultured in a humidified incubator at 37°C in 5% CO_2_. Cell culture media DMEM and RPMI1640 were purchased from PanEco (Moscow, Russia). Fetal bovine serum was from HyClone (Cramlington, United Kingdom).

### Reagents

Recombinant proteins TRAIL (amino acid residues 114–280) and its DR5-selective mutant variant DR5-B were expressed in *Escherichia coli* and purified in our laboratory as previously described (Yagolovich et al., 2019). Brefeldin A and cycloheximide were purchased from Tocris (Bristol, United Kingdom). Pan-caspase inhibitor Z-VAD-FMK was from Santa Cruz Biotechnology (Dallas, TX, United States).

### Cell Viability Assay

A549, HT-29, and HCT116 cells were seeded in 96-well plates at a density of 1 × 10^4^ per well in 100 μl culture medium and incubated for 24 h in humidified atmosphere of 5% CO_2_ (New Brunswick, Eppendorf, Germany) at 37°C. The culture medium was aspirated and 100 μl of fresh serum free medium supplemented with TRAIL or DR5-B was added to the wells. In the case of Jurkat, cells were harvested, washed with serum-free medium and plated in 96-well plates (5 × 10^4^ cells per well) in 100 μl of culture medium without serum and 50 μl of TRAIL or DR5-B solutions at the appropriate concentrations were added to each well. The cells were incubated for 24 h, 10 μl of water soluble tetrazolium salts reagent (WST-1) (Sigma-Aldrich, St. Louis, MO, United States) was added to each well and incubation was continued for another 2 h at 37°C. The WST-1 assay is based on the cleavage of the tetrazolium salt WST-1 to formazan by cellular mitochondrial dehydrogenases. Viable cells have a high activity of mitochondrial dehydrogenases, which leads to the formation of the dye formazan. The optical density of the wells was measured using an iMark plate spectrophotometer (Bio-Rad, United States) at a wavelength of 450 nm with background subtraction at 655 nm.

### Flow Cytometry

The assays were performed as described earlier, with some modifications (Artykov et al., 2020).

The cells were seeded in 6 well plate at a density of 2 × 10^5^ cells per well in 2 ml of culture media and incubated for 24 h in humidified atmosphere of 5% CO_2_ at 37°C. After washing with serum-free medium, the cells were incubated with TRAIL or DR5-B for the indicated time (5–60 min or 1–24 h). Cells were detached from the culture flasks with Versene solution, washed with ice-cold PBS, and resuspended in FACS buffer (PBS with 1% BSA). Cell suspensions were incubated for 1 h at 4°C with 5 μg/ml anti-DR4 (DR-4-02) or anti-DR5 (DR5-01-1) monoclonal antibodies (GeneTex, Irvine, CA, United States). Next the cells were washed twice and incubated with 20 μg/ml secondary antibodies Alexa Fluor 488 (Invitrogen, Waltham, MA, United States) for 1 h at 4°C, washed twice, and suspended in FACS buffer supplemented with propidium iodide. Mouse IgG1 (15H6, Genetex) was used as an isotype control. The cell surface expression of DR4 and DR5 was analyzed on a CytoFlex flow cytometer (Beckman Coulter, Brea, IN, United States).

### Confocal Microscopy Analysis

Glass slides were placed in 6 well plates and 2 × 10^5^ cells were seeded in each well. Cells were cultured in 2 ml of culture media and incubated for 24 h. After washing with serum-free medium cells were treated with 100 ng/ml or 1,000 ng/ml of TRAIL variants. At the indicated times, the medium was aspirated and the dishes were transferred to ice and washed with cold PBS. Subsequently, cells were fixed in 3% paraformaldehyde for 20 min. In order to analyze the expression of receptors only on the surface of the plasma membrane, the permeabilization step was skipped. After washing with ice-cold PBS and blocking in 3% BSA in PBS for 30 min, the primary antibodies to death receptors DR5 (DR5-01-1) and DR4 (DR-4-02) were added at a concentration of 2 μg/ml and the cells were incubated for 1 h at room temperature. The slides were washed three times with PBS and incubated with 4 μg/ml goat anti-mouse IgG Alexa Fluor 488 and Hoechst 33342 in the dark for 1 h at room temperature. The confocal LSM analysis was performed on Leica TCS SPE (Leica microsystems, Wetzlar, Germany) equipped with immersion ×100 objective with a 1.4 numerical aperture.

### Statistical Analysis

Statistical analyses for each experiment were performed as described in the corresponding figure legends. Multiple comparison analyses for one-way ANOVA followed by Tukey test or followed by Dunnett’s *post hoc* test were performed using GraphPad Prism 8. Experimental phenotypes were confirmed in at least three independent experiments.

## Results

### TRAIL Decreases the Surface Expression of DR4 and DR5 Receptors in a Concentration-Dependent Manner

**FIGURE 1:**
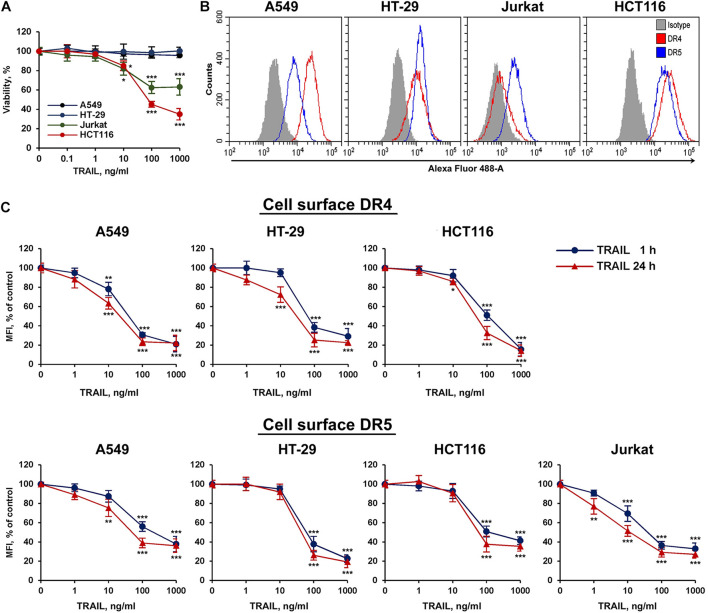
TRAIL decreased surface expression of DR4 and DR5 receptors in concentration dependent manner. **(A)** Viability of HT-29, A549, HCT116, and Jurkat cells after TRAIL treatment for 24 h determined by WST-1 colorimetric assay. **(B)** Cell surface expression of TRAIL death receptors determined by flow cytometry. **(C)** Cells were treated with TRAIL in indicated concentrations for 1 or 24 h and surface expression of DR4 and DR5 was determined by flow cytometry. The data represent means ± SDs of triplicate assays. *p < 0.05, **p < 0.01, and ***P < 0.001 indicate significant difference from the control according to One-way ANOVA followed by Dunnett’s *post hoc* test.

To study TRAIL-stimulated traffic of death receptors DR4 and DR5, two sensitive (HCT116 and Jurkat) and two resistant (HT-29 and A549) tumor cell lines were selected (Figure 1A). All cell lines expressed a comparable amount of death receptors on the cell surface, except that the level of the DR4 receptor was practically undetectable in Jurkat cells (Figure 1B). The absence of the surface expression of DR4 on Jurkat cells has been shown earlier in several studies (Jang et al., 2003; Merino et al., 2006). Thus, the traffic of this receptor in Jurkat cells was not investigated in the further experiments. To analyze possible competition for receptor binding between TRAIL and antibodies, we used recombinant DR5 and DR4 extracellular domains captured at plates. The binding of anti-DR antibodies analyzed by ELISA (enzyme-linked immunosorbent assay) was not affected by TRAIL, indicating no competition (Supplementary Figure 1 and Supplementary Table 1). In all cell lines, a decrease in surface expression of death receptors was measured during 1 and 24 h upon stimulation with TRAIL in a concentration-dependent manner (Figure 1C). Significant decrease of surface receptor expression was detected at TRAIL concentration of more than 10 ng/ml. The exception was Jurkat cells, where the effect was observed at lower concentrations of TRAIL probably because the ligand was not titrated by the DR4 receptor. The level of receptors on the cell surface did not differ significantly after incubation 1 or 24 h with high concentration of TRAIL (1,000 ng/ml), while at lower concentrations of ligand the number of receptors remaining on the plasma membrane was slightly lower after 24 h. These data indicated that TRAIL mediated downregulation of surface DRs in time- and concentration- dependent manner. It should be noted that the concentration of endogenous soluble TRAIL in blood is approximately 0.1–1 ng/ml, which is insufficient to stimulate the significant decrease of surface receptor exposure (Cheng et al., 2015). Thus, the possible physiological role of downregulation the surface expression of DR4 and DR5 upon stimulation of higher TRAIL concentrations currently remain unclear. To our knowledge, there are no publications describing a dramatic increase in the concentration of TRAIL under physiological conditions. The concentration of TRAIL in the blood rises sharply in clinical trials using high doses of the drug (5–20 mg/kg). According to pharmacokinetic profiles, after 1 h of drug administration, the concentration of TRAIL in the blood of patients was 20–150 ng/ml, depending on the dosage (Soria et al., 2010). Therefore, when treating the neoplastic diseases with TRAIL, it is extremely important to consider the effect of death receptor traffic on drug efficacy. In addition, numerous studies have shown that the surface expression of TRAIL DRs is upregulated under the influence of various chemotherapeutic and natural agents, what in turn can reduce the concentration of the ligand required to induce their internalization.

### TRAIL Mediates Decrease of Surface Death Receptors With Similar Rate in Resistant and Sensitive Cell Lines

**FIGURE 2:**
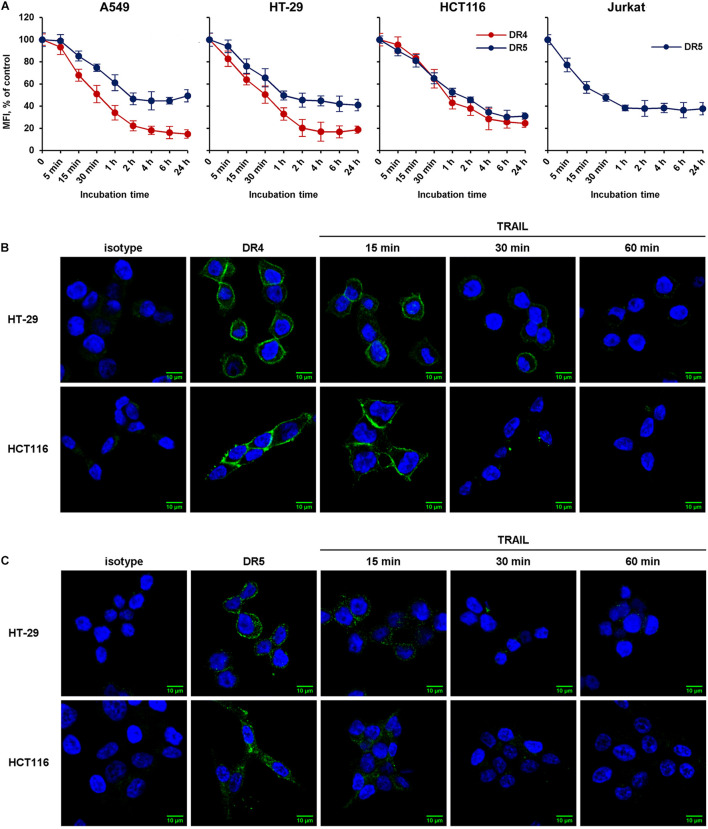
The rate of TRAIL-mediated downregulation of surface DR4 and DR5 in resistant and sensitive tumor cell lines. **(A)** TRAIL-resistant (HT-29, A549) and TRAIL-sensitive (HCT116, Jurkat) cells were treated with 100 ng/ml TRAIL and surface expression of DR4 and DR5 was determined at indicated times by flow cytometry. The data represent means ± SDs of triplicate assays. Immunofluorescence staining of DR4 **(B)** and DR5 **(C)** receptors in HT-29 and HCT116 cells before and after TRAIL treatment analyzed by confocal LSM. To detect receptors only on the plasma membrane, the permeabilization step was excluded during samples preparation.

We then compared the kinetics of TRAIL-induced downregulation of the DR4 and DR5 receptors in sensitive (HCT116 and Jurkat) and resistant (A549 and HT-29) cell lines (Figure 2A). Cells were incubated with 100 ng/ml TRAIL for 5, 15, 30 min and then from 1 to 24 h, and the surface expression of receptors was determined by flow cytometry. According to the data, the bulk of both receptors were internalized within 1 h in all cell lines. One hour later, the process slowed down and after 2 h reached an equilibrium, in which the values practically did not change during 24 h. The flow cytometry data were confirmed by confocal microscopy analysis. To detect receptors only on the plasma membrane, the permeabilization step was excluded during samples preparation (Figures 2B,C). Obtained data clearly indicated that TRAIL induced decrease of DR4 and DR5 exposure in both TRAIL-sensitive HCT116 and TRAIL-resistant HT-29 cells with similar efficiency. Thus in both sensitive and resistant lines TRAIL does induce internalization despite the differences in the phenotype—apoptosis induction.

### DR5-Selective TRAIL Variant DR5-B Internalizes Only DR5 Receptor

**FIGURE 3:**
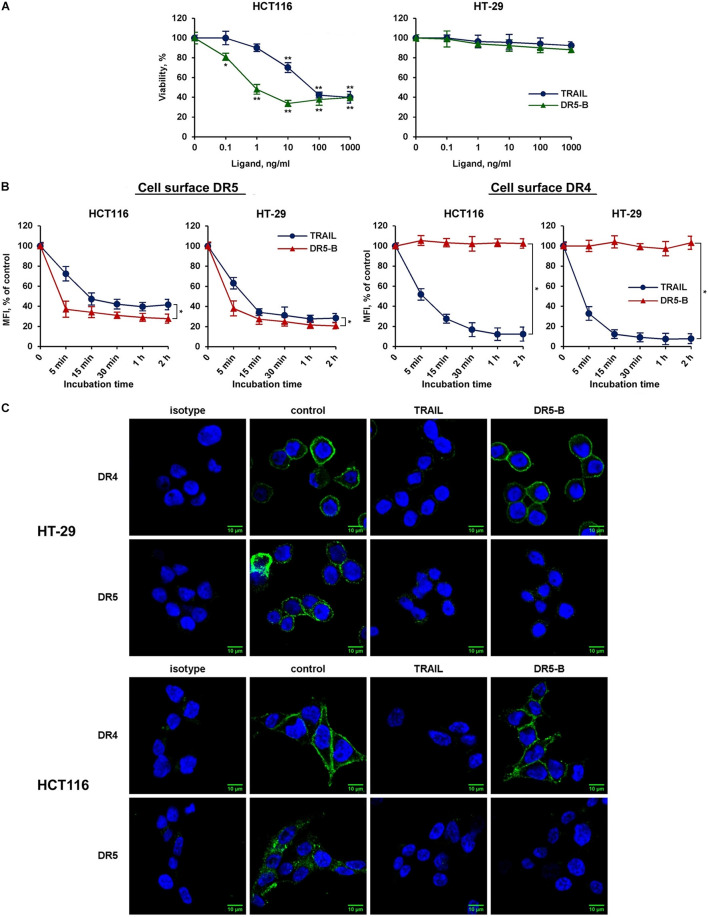
DR5-selective variant of TRAIL DR5-B induced the decrease of surface expression of DR5 but not of DR4 receptor. **(A)** Viability of HT-29 and HCT116 cells after treatment with TRAIL or DR5-B at the indicated concentrations for 24 h as determined by the WST-1 colorimetric assay. The data represent means ± SDs of triplicate assays. *p < 0.01 and **p < 0.001 indicate significant difference from the control according to One-way ANOVA followed by Dunnett’s *post hoc* test. **(B)** TRAIL-resistant HT-29 and TRAIL-sensitive HCT116 cells were treated with 1,000 ng/ml TRAIL or DR5-B and the surface expression of DR4 and DR5 was determined as shown in Figure 2. The data represent means ± SDs of triplicate assays. *p < 0.001 indicate significant difference between groups according to Two-way ANOVA. **(C)** Immunofluorescence staining of the DR4 and DR5 receptors in HT-29 and HCT116 cells before and after TRAIL treatment analyzed by confocal LSM. To detect receptors only on the plasma membrane, the permeabilization step was excluded during samples preparation.

To elucidate the possible interaction of death receptors during internalization, a DR5-selective TRAIL variant was used. We have previously designed and purified TRAIL mutant variant DR5-B that selectively binds to DR5 receptor and lacks the affinity to DR4 and to decoy receptors DcR1 and DcR2 (Gasparian et al., 2009). TRAIL- and DR5-B-sensitive (HCT116) and resistant (HT-29) cells (Figure 3A) were incubated with ligands at a concentration of 1,000 ng/ml, and the time-dependent decrease of DR4 and DR5 surface expression was determined (Figure 3B). TRAIL and DR5-B stimulated the internalization of the DR5 receptor in both cell lines, but DR5-B worked faster and more efficiently. The rate of internalization as well as the absolute amount of internalized molecules was higher when cells were treated with DR5-B. After 5 min of incubation of HCT116 cells with DR5-B, 68% of DR5 was internalized, while TRAIL reduced the amount of this receptor by only 26%. The similar results were obtained in HT-29 cells. This is apparently due to the fact that TRAIL is titrated by the other receptors (DR4 or decoy receptors) as the dissociation constants of TRAIL and DR5-B to the DR5 receptor, determined earlier using surface plasmon resonance, practically did not differ (0.51 × 10^–9^ M and 0.71 × 10^–9^ M, respectively) (Gasparian et al., 2009). In contrast, the DR4 surface expression was not affected by DR5-B, whereas TRAIL reduced it by 90% in both lines. These data were confirmed by the confocal microscopy measurements of receptor exposure on the plasma membrane (Figure 3C). Our results are in good agreement with the data obtained in the work of Nahacka et al. (2018), where the authors compared the composition of DISC (death inducing signaling complex) formed by different DR-selective mutant variants of TRAIL. It was demonstrated that the DISC formed by DR5-B did not contain DR4 receptor in the HT-29 and PANC-1 cell lines, while in the DISC formed by TRAIL both DRs were detected. However, DR4 internalization by DR5-B was observed earlier after strong upregulation of the surface DRs by chemotherapeutic agents (Artykov et al., 2020). Obviously, increased expression of death receptors on the cell surface promotes the formation of heterodimers where the receptors can be internalized together as part of the same DISC (Szegezdi et al., 2012). Based on the obtained data, it can be assumed that DR5 can be internalized independently of DR4 receptor.

### TRAIL Did Not Affect the Steady-State Expression of DR4 and DR5 in Cell Lysates

**FIGURE 4:**
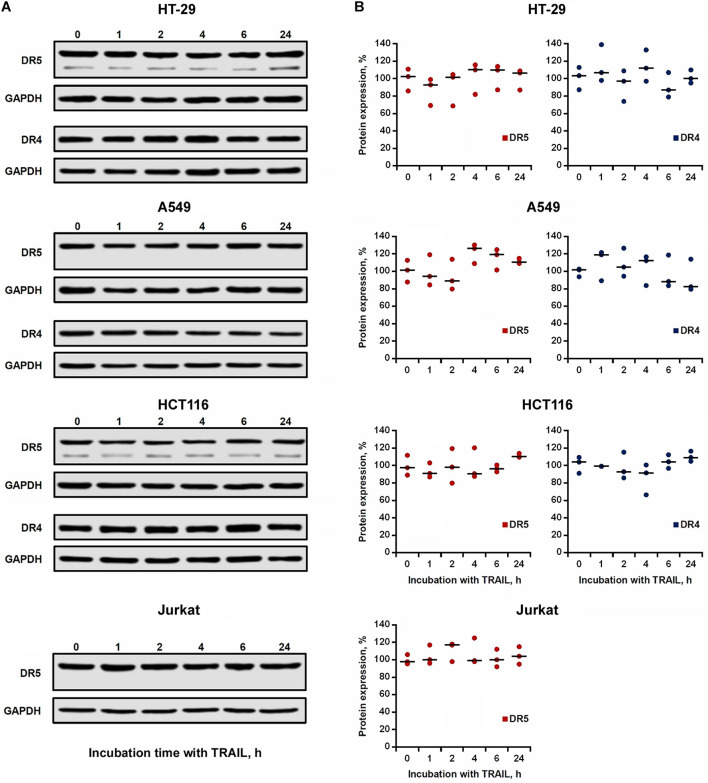
Expression of DR4 and DR5 in cell lysates remained relatively stable after TRAIL treatment. **(A)** HT-29, A549, HCT116 and Jurkat cells were treated with 1,000 ng/ml TRAIL for 1, 2, 4, 6, and 24 h, and the expression of death receptors in cell lysates was analyzed by Western blotting. **(B)** Protein band intensities was calculated using the ImageJ software (http://rsbweb.nih.gov/ij/, NIH, Bethesda, MD, United States) and data were normalized to GAPDH. Data are expressed as the means ± SD of three independent experiments. The Dunnett’s multiple comparisons tests following one-way ANOVA did not find a significant difference among means.

We then investigated the effect of TRAIL on the total expression of DR4 and DR5 receptors in cell lysates. TRAIL-sensitive (HCT116 and Jurkat) and TRAIL-resistant (HT-29 and A549) cells were incubated with 1,000 ng/ml TRAIL for 1, 2, 4, 6, and 24 h, and the receptors content was analyzed by Western blotting with monoclonal antibodies to DR4 and DR5 (Figure 4A). In all tested cells, we did not register significant changes in both DR4 and DR5 contents during the entire incubation period with TRAIL (Figure 4B). Usually the endocytosed receptors are shuttled to early endosomes, sorted to late endosomes and finally to the lysosomes for degradation. In the event when the cell is re-sensitized with a stimulatory agent, the receptor travels back to the plasma membrane directly or through recycling endosomes. Post-endocytic localization and trafficking of the TRAIL death receptors are poorly investigated. It has been shown that DR4 and DR5 receptors, after TRAIL stimulation, are transported from the plasma membrane to the nucleus or can co-localize with endosomes or lysosomes (Zhang et al., 2000; Akazawa et al., 2009; Mert et al., 2019). In any case, evidently during the incubation of cells with TRAIL, the rate of the supposed receptor degradation is comparable to the rate of synthesis of new molecules, and, therefore, the total level of protein in the cells remains stable.

### Brefeldin A Inhibited the Recovery of Surface DR4 and DR5

**FIGURE 5:**
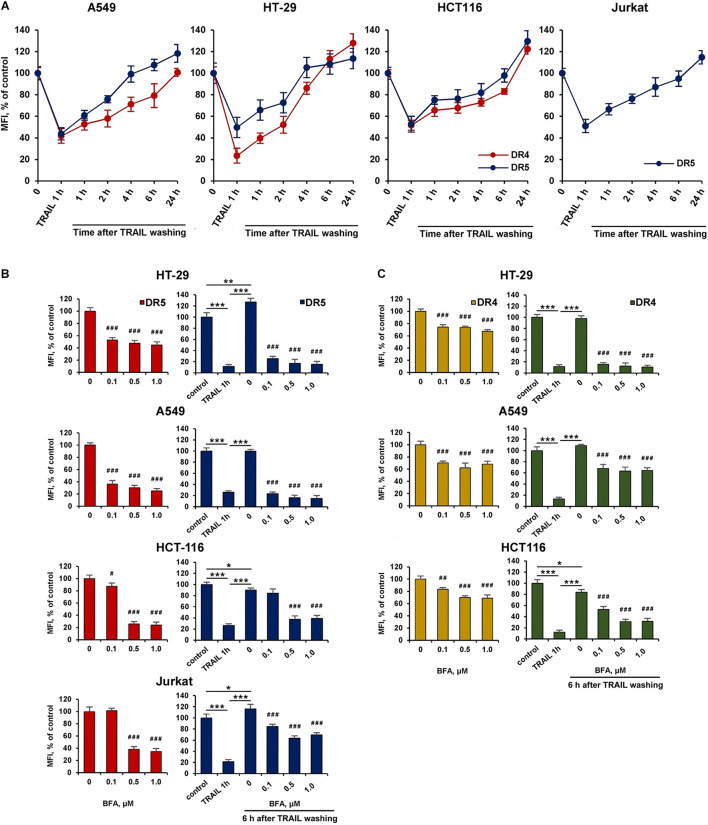
Brefeldin A inhibited the recovery of surface DR4 and DR5 receptors. **(A)** HT-29, A549, HCT116, and Jurkat cells were treated with 1,000 ng/ml TRAIL for 1 h, the ligand was washed three times with ice-cold medium and the kinetics of surface receptor recovery was analyzed for 24 h by flow cytometry. Mean Fluorescence Intensity (MFI) values are presented as a percentage relative to control cells. Data represent means ± standard deviation of three analyzes. **(B)** Cells treated with Brefeldin A (BFA) before or after TRAIL (1 μg/ml) treatment at indicated concentrations for 6 h and the surface expression of DR5 **(B)** and DR4 **(C)** was determined by flow cytometry. Mean Fluorescence Intensity (MFI) values are presented as a percentage relative to BFA non-treated cells. Data represent means ± SD of three independent experiments. *p < 0.05, **p < 0.01, and ***p < 0.001 indicate significant difference between groups according to One-way ANOVA followed by Tukey test. ^#^*p* < 0.05, ^##^*p* < 0.01, and ^###^*p* < 0.001 indicated significant difference from the untreated with BFA cells according to One-way ANOVA followed by Dunnett’s *post hoc* test. Raw data for **(B,C)** are available in Supplementary Table 3.

We then investigated the kinetics of cell surface receptor recovery after TRAIL-induced endocytosis. For this, cells were incubated with TRAIL for 1 h, the ligand was washed off, and the surface expression of receptors was determined for 1, 2, 4, 6, and 24 h (Figure 5A). After removing the ligand from the culture medium, the amount of surface DR4 and DR5 increased slowly and after 4 h (in HT-29 and A549 cells) and 6 h (in HCT116 and Jurkat cells) reached values corresponding to that of untreated cells. It remains unclear whether faster DRs recovery plays an important role in cell resistance. Additional experiments are needed to verify this phenomenon. Interestingly, after prolonged (24 h) incubation, the number of receptors on the cell surface was even higher (20–30%) compared to TRAIL-untreated cells indicating that TRAIL promoted the upregulation of its death receptors surface expression. Brefeldin A (BFA), an potent ER stressor, which destroys Golgi compartments and depletes the delivery of substances to the cell surface from secretory pathway, significantly decreased DR5 and DR4 surface expression in time- (Supplementary Figure 2 and Supplementary Table 2) and concentration-dependent manner in all cell lines (Figures 5B,C and Supplementary Table 3). Obtained data indicate that TRAIL receptors undergo spontaneous ligand-independent internalization regardless of the cell sensitivity. The recovery of surface DR4 and DR5 after TRAIL-stimulated endocytosis was also strongly inhibited by BFA in all cell lines. In addition, receptor surface levels were comparable after BFA treatment with and without TRAIL. These data indeed show the importance of the Golgi apparatus and indicate the existence of constitutive endocytosis of DRs. It was earlier shown that BFA leads to TRAIL DRs accumulation in the Golgi apparatus, suggesting that this organelle forms a platform for DR signaling in stressed cells (van Raam et al., 2017). Recently it was demonstrated that endoplasmic reticulum (ER) stress initiated apoptosis through intracellular activation of DR5 independently of TRAIL and that misfolded proteins can directly engage with DR5 in the ER-Golgi intermediate compartment, where DR5 assembles pro-apoptotic caspase 8-activating complexes (Lam et al., 2020). Thus, the Golgi apparatus may be involved in the signaling of the post-endocytic DR4 and DR5 receptors.

### Cell-Type Specific Action of Cycloheximide on the Expression and Recovery of Surface the DR4 and DR5

**FIGURE 6:**
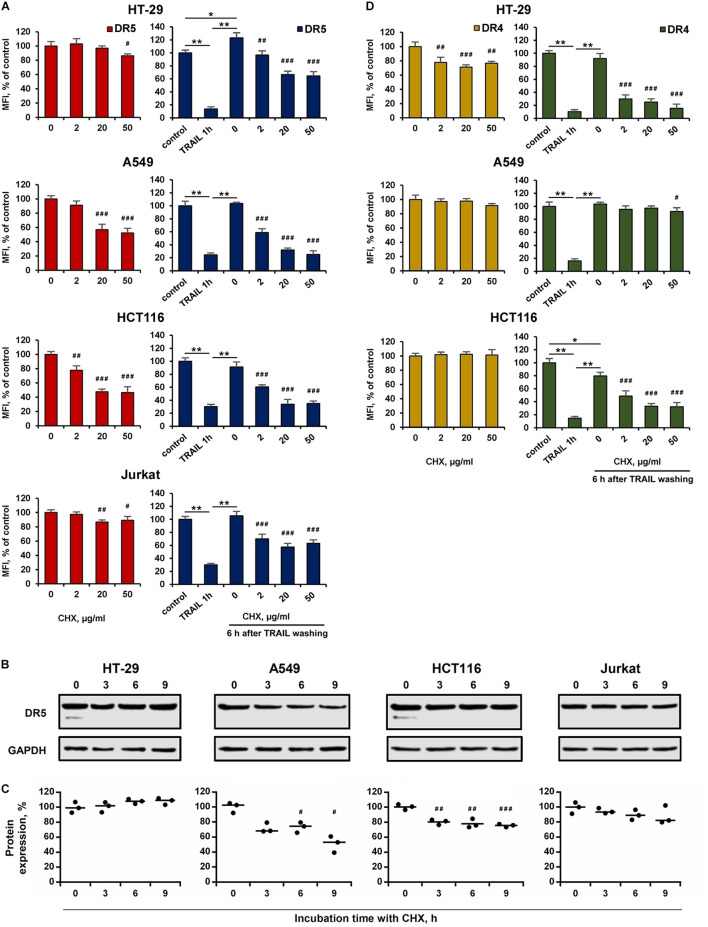
Effect of cycloheximide on expression and recovery of surface DR4 and DR5. HT-29, A549, HCT116 and Jurkat cells were treated with cycloheximide (CHX) before or after TRAIL (1 μg/ml) treatment at indicated concentrations for 6 h and the surface expression of DR5 **(A)** and DR4 **(D)** was determined by flow cytometry. Mean Fluorescence Intensity (MFI) values are presented as a percentage relative to CHX non-treated cells. **(B)** Western blot analysis of DR5 receptor in cell lysates after treatment with 50 µg/ml CHX for 3, 6 and 9 h. **(C)** Protein band intensities calculated using the ImageJ software (http://rsbweb.nih.gov/ij/, NIH, Bethesda, MD, United States) and data were normalized to GAPDH. Data are expressed as the means ± SD of three independent experiments. Mean Fluorescence Intensity (MFI) values are presented as a percentage relative to BFA non-treated cells. Data represent means ± SD of three independent experiments. *p < 0.01 and **p < 0.001 indicate significant difference between groups to One-way ANOVA followed by Tukey test. ^#^*p* < 0.05, ^##^*p* < 0.01 and ^###^*p* < 0.001 indicated significant difference from the untreated with CHX cells according to One-way ANOVA followed by Dunnett’s posthoc test. Raw data for **(A,D)** are available in Supplementary Table 4.

The effect of BFA on the receptor surface expression was unambiguous for all cell lines before and after TRAIL treatment (Figures 5B,C and Supplementary Table 3). However, the protein synthesis inhibitor cycloheximide (CHX) affected differently the expression of surface DRs in various cell types. CHX significantly inhibited both surface and total DR5 expression in A549 and HCT116 cells, whereas this effect was negligible in HT-29 and Jurkat cells (Figures 6A–C and Supplementary Table 4). Accordingly, after TRAIL-stimulated endocytosis, the surface restoration of this receptor was completely blocked in A549 and HCT116, but in HT-29 and Jurkat cells, it was only partially inhibited by CHX. The surface DR4 was virtually unaffected by CHX in HCT116 and A549 cells and decreased by about 20% in HT-29 cells (Figure 6D and Supplementary Table 4). However, the DR4 recovery after TRAIL stimulation was completely inhibited in HCT116 and HT-29 cells. Interestingly, CHX did not affect either DR4 surface expression in general or its recovery after TRAIL stimulation in A549 cells, indicating that DR4 recovery occurred from the intracellular compartments where it could be accumulated. Thus, the results of the experiment using CHX showed that there is no correlation between the sensitivity of cells to TRAIL and the balance between degradation, synthesis and recycling of receptors. Despite the fact that the effect of two inhibitors (BFA and CHX) was aimed at reducing the number of surface receptors, the inhibitory effect of BFA was more potent and universal either with or without TRAIL. Comparing the results in Figures 5, 6, it can be seen that there are significant differences in the effects of BFA and CHX on the DRs surface expression. BFA significantly (by 60–70%) reduced the amount of DR5 on the surface of all tested cell lines, while CHX acted only on A549 and HCT116. In lines A549 and HCT116, the surface expression of DR4 was decreased by BFA, but not by CHX. These data indicated the important role of the Golgi apparatus in the restoration of post-endocytic receptors. Thus, it can be concluded that post-endocytic receptor recovery can occur not only from newly synthesized molecules, but also from the intracellular compartments, in particular from TGN. The inhibitory effects of BFA and CHX on the restoration of receptor surface expression indicated that TRAIL death receptors are continuously synthesized, externalized, internalized and degraded, and these processes are more pronounced for DR5 receptor.

### Inhibition of Receptors Endocytosis by Hypertonic Sucrose Sensitized the Resistant Cells to TRAIL

**FIGURE 7:**
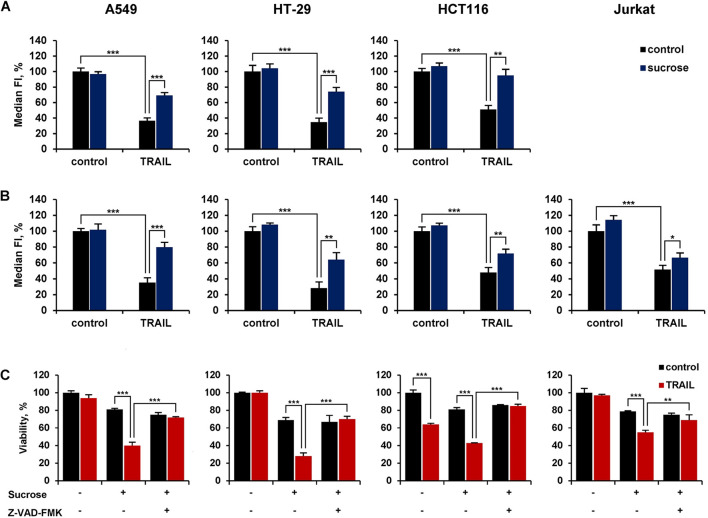
Hypertonic sucrose inhibited TRAIL-mediated receptor endocytosis and sensitized resistant cells to TRAIL. HT-29, A549, HCT116 and Jurkat cells were treated with or without 250 mM sucrose for 1 h followed by treatment with 100 ng/ml TRAIL for another 1 h and the surface expression of DR4 **(A)** and DR5 **(B)** was determined by flow cytometry. **(C)** Cells were incubated with or without 250 mM sucrose for 1 h in the absence or presence of 10 μM Z-VAD-FMK followed by treatment with 100 ng/ml TRAIL for 16 h in the case of HT-29 and A549 cells, 7 h for HCT116 and 3 h for Jurkat cells. Viability was determined by WST-1 colorimetric assay. The results are presented as mean ± SD of triplicate independent experiments. *p < 0.05, **p < 0.01 and ***p < 0.001 indicate significant difference between groups according to One-way ANOVA followed by Tukey test. Raw data are available for **(A,B)** in Supplementary Table 6 and for **(C)** in Supplementary Table 7.

We then investigated the role of ligand-mediated endocytosis of DR4 and DR5 on the cytotoxic activity of TRAIL. In our hands, the inhibitor of clathrin-mediated endocytosis dynasore or cholesterol-depleting agent filipin III did not significantly inhibit TRAIL-mediated endocytosis of DR4 or DR5 (Supplementary Figure 3 and Supplementary Table 5). The hypertonic sucrose is known to non-selectively block the receptor endocytosis (Guo et al., 2015). TRAIL-mediated endocytosis of DR4 and DR5 was inhibited when cells were pretreated with sucrose at concentration 250 mM for 1 h (Figures 7A,B and Supplementary Table 6). In addition, A549 and HT-29 resistant cells were effectively sensitized to TRAIL when pre-incubated with 250 mM sucrose (Figure 7C and Supplementary Table 7). The cytotoxicity of TRAIL was also increased in HCT116 and Jurkat cells in the presence of sucrose. Hyperosmotic sucrose was highly cytotoxic to Jurkat cells during prolonged exposure (data not shown). Therefore, these cells were incubated with TRAIL for 3 h, which is insufficient to manifest the cytotoxic activity of TRAIL. Nevertheless, upon treatment of these cells with sucrose, a decrease in cell viability by TRAIL was observed in 3 h. The general caspase inhibitor z-VAD-FMK (10 μM) completely blocked the increased cytotoxic activity of TRAIL observed after incubation of cells in a hyperosmotic state, demonstrating that the decrease in cell viability is a result of the apoptotic mechanism activation induced by TRAIL but not by sucrose as hyperosmolarity itself did not induce apoptosis. Thus, the inhibition of TRAIL death receptor endocytosis by sucrose enhances the cytotoxic activity of TRAIL, suggesting that endocytosis is a defense mechanism for cell survival.

## Discussion

The cytokine TRAIL induces apoptosis through the death receptors DR4 or DR5, predominantly in cancer cells, but not in normal cells (Wajant, 2019). However, many cancer cells are resistant to DRs-mediated apoptosis due to a variety of mechanisms, and this is the reason for the low antitumor activity of its various therapeutic agonists (recombinant TRAIL variants or antibodies to receptors) in clinical trials (Micheau et al., 2013; Kretz et al., 2019). Although multiple proteins are involved in DR-mediated apoptosis, surface expression of death receptors is a prerequisite for the activation of TRAIL apoptosis signaling. Expression of DR4 and DR5 receptors is regulated at the transcriptional level by epigenetic modification, transcription factors, microRNA and RNA-binding proteins, as well as at post-translational level by ubiquitination and glycosylation (Song et al., 2010; van de Kooij et al., 2013; Micheau, 2018; Min et al., 2019). However, a high level of surface receptor expression does not always correlate with the sensitivity to TRAIL (Chen et al., 2012). Numerous studies have demonstrated that DRs can be located in various cellular compartments such as autophagosomes, trans-Golgi network, and nucleus or even in the cytosol (Bertsch et al., 2014). The mechanisms of DRs expression and signaling have been extensively studied in the last two decades, but little research has focused on the regulation of their membrane transport.

In the present study, we compared the kinetics of TRAIL-mediated decrease of surface DR4 and DR5 receptors expression in TRAIL-resistant (HT-29, A549) and TRAIL-sensitive (HCT116, Jurkat) tumor cell lines. Both receptors surface expression was rapidly decreased after TRAIL binding in a concentration-dependent manner with similar kinetics in all tested cell lines. TRAIL-mediated rapid internalization of DR5 in Colo205 cells (Austin et al., 2006) or rapid internalization of TRAIL itself in BJAB, Hela (Kohlhaas et al., 2007), MDA-MB-231 and A549 (Zhang et al., 2009; Reis et al., 2017) and Huh-7 (Akazawa et al., 2009) cells have been described earlier. We measured for the first time the kinetics of TRAIL-mediated decrease in surface DR4 simultaneously with DR5 and showed that both receptors were internalized at the same rate. The receptor-selective TRAIL variant DR5-B decreased only surface DR5 but not DR4 indicating that death receptors can be internalized independently of each other. We did not observe changes to total death receptor levels during incubation of cells with TRAIL, since steady-state expression of DR4 and DR5 in cell lysates remained unchanged, possibly because the rate of putative degradation was equilibrated with the rate of synthesis of new molecules.

The recycling of DR4 and DR5 back to the plasma membrane after endocytosis was not investigated until now. Here we have demonstrated that both receptors slowly return to the plasma membrane after TRAIL washing from culture medium and within 6 h the surface expression of receptors was completely restored, regardless of the sensitivity of the tumor cells to TRAIL-induced apoptosis. The slow recycling pathway involves the transport of cargo proteins from the early endosome to the endocytic recycling compartment (ERC) and from the ERC to the plasma membrane (Grant and Donaldson, 2009). The recovery of surface DR4 and DR5 was blocked by Golgi-disrupting agent BFA and partially suppressed by protein synthesis inhibitor CHX. Since TRAIL did not affect the stable expression of DRs in cell lysates. it can be assumed that the recovery of surface receptors can occur both from ERC (late endosomes and Golgi apparatus, or nucleus), where they accumulate, and from the newly synthesized molecules. It has recently been demonstrated that nuclear TRAIL-DRs are directly translocated from the plasma membrane through an initial clathrin-dependent endocytosis in a TRAIL-dependent manner, independently of its apoptotic activity (Mert et al., 2019). More research is needed to clarify how DRs endocytosis correlates with their recovery and how these processes are regulated.

We have also shown that TRAIL death receptors undergo constitutive endocytosis in the absence of a ligand. BFA decreased surface expression of both receptors in time- (1–6 h) and concentration-dependent manner, and this effect was more pronounced for DR5. The effect of brefeldin A was not associated with receptor degradation, since the content of DR5 in HT-29 and Jurkat cells or surface expression of DR4 in A549 and HCT116 cells were not affected by cycloheximide. The secretory stressors such as BFA and thapsigargin (Tg) have been shown to induce accumulation of death receptors in the Golgi apparatus and its compensatory expression (Lu et al., 2014; van Raam et al., 2017). Despite its importance, the details of TRAIL death receptors endosomal traffic have not been investigated. The signal recognition particle complex (SRP) is required for DR4, but not DR5 localization on the cell surface, indicating that receptors transport may be regulated in different ways (Ren et al., 2004). Recently it has been demonstrated that depletion of MLKL (Mixed lineage kinase domain-like) reduced the endosomal traffic and degradation of DR5, resulting in increased TRAIL cytotoxicity (Park et al., 2020). A deeper understanding of the molecular mechanisms that support DRs transport along the recycling pathway will provide a deeper understanding of the mechanisms of resistance of tumor cells to TRAIL and, probably, will determine new approaches to the treatment of tumor diseases.

Several studies have shown that the disruption of clathrin-dependent endocytosis of DRs by inactivation of dynamins (particularly by dynamin 1) leads to increased cell apoptosis (Austin et al., 2006; Reis et al., 2017). We did not observe any effect of the inhibitor of clathrin-mediated endocytosis dynasore on TRAIL-mediated DR4 or DR5 endocytosis. Dynasore is a cell-permeable inhibitor of dynamin GTPase activity that leads to the accumulation of late invaginated coated pits (Nankoe and Sever, 2006). One of the possible reasons for the lack of dynasore effect may be that antibodies to DRs cannot recognize receptors when they are in the O-shaped pits. Hyperosmotic sucrose blocks formation of type 1 coated pits by preventing clathrin and adaptors from interacting (Hansen et al., 1993). Under such conditions, the availability of receptors for antibodies is not impaired, and this is probably why we observed inhibition of TRAIL-mediated decrease in surface DRs upon pretreatment of cells with hyperosmotic sucrose. Resistant A549 and HT-29 cells were effectively sensitized to TRAIL-induced cell death under sucrose hyper-osmosis. Several studies have demonstrated that DR internalization is not required for the formation of the death inducing signaling complex (DISC) or for apoptosis (Kohlhaas et al., 2007; Zhang et al., 2009). However, the reasons for DISC inactivation after endocytosis remain unclear. In our experiments, the kinetics of receptor internalization practically did not differ in resistant and sensitive cells. However, the rate of DRs recovery in sensitive cells was relatively reduced. It can be assumed that the DISC components dissociate relatively faster after internalization in TRAIL-resistant cells, preventing the initiation of apoptosis.

Thus, we have demonstrated that the sensitivity of tumor cells is not related to the rate of TRAIL-mediated DR endocytosis. Based on our results it can be proposed that the post-endocytic events, such as the rate of DISC dissociation and accumulation of receptors in different compartments, or the rate of their degradation play a significant role in triggering apoptotic TRAIL signaling. Additional experimental data are needed to confirm this hypothesis.

## Data Availability Statement

The original contributions presented in the study are included in the article/Supplementary Material, further inquiries can be directed to the corresponding author/s.

## Author Contributions

AA, AY, MG, and DT performed the experiments and analyzed the data. DD and MK designed the research. MG wrote the manuscript. DD carefully revised the manuscript. All authors have read and approved the final manuscript.

## Conflict of Interest

The authors declare that the research was conducted in the absence of any commercial or financial relationships that could be construed as a potential conflict of interest.

## Publisher’s Note

All claims expressed in this article are solely those of the authors and do not necessarily represent those of their affiliated organizations, or those of the publisher, the editors and the reviewers. Any product that may be evaluated in this article, or claim that may be made by its manufacturer, is not guaranteed or endorsed by the publisher.
