# Corrigendum: Death Receptors DR4 and DR5 Undergo Spontaneous and Ligand-Mediated Endocytosis and Recycling Regardless of the Sensitivity of Cancer Cells to TRAIL

**DOI:** 10.3389/fcell.2021.820069

**Published:** 2022-02-14

**Authors:** Artem A. Artykov, Anne V. Yagolovich, Dmitry A. Dolgikh, Mikhail P. Kirpichnikov, Daria B. Trushina, Marine E. Gasparian

**Affiliations:** ^1^ Department of Bioengineering, Institute of Bioorganic Chemistry (RAS), Moscow, Russia; ^2^ Faculty of Biology, Lomonosov Moscow State University, Moscow, Russia; ^3^ Department of X-Ray and Synchrotron Research, A.V. Shubnikov Institute of Crystallography of Federal Scientific Research Centre “Crystallography and Photonics” of Russian Academy of Sciences, Moscow, Russia

**Keywords:** DR5, DR4, receptor endocytosis, receptor recycling, membrane traffic, brefeldin A

In the original article there was an error in the **Funding statement**. The statement was missing a link for the funder “Russian Science Foundation.” The corrected Funding statement appears below.

This work was supported by the Russian Science Foundation (Grant No. 21-14-00224, https://rscf.ru/project/21-14-00224/; AA, MG, and DD production of recombinant TRAIL and DR5-B, TRAIL-mediated receptors traffic), by budgetary funding, AY, MK for western blot and ELISA analysis and of Lomonosov Moscow State University (Moscow, Russia). The confocal laser scanning microscopy study DT was performed using the equipment of the Shared Research Center FSRC “Crystallography and Photonics” RAS and supported by the Ministry of Science and Higher Education of the Russian Federation within the State assignment FSRC “Crystallography and Photonics” RAS.

The authors apologize for this error and state that this does not change the scientific conclusions of the article in any way. The original article has been updated.

