# Supplementary material for: Death Receptors DR4 and DR5 Undergo Spontaneous and Ligand-Mediated Endocytosis and Recycling Regardless of the Sensitivity of Cancer Cells to TRAIL
Source: Front Cell Dev Biol. 2021 Sep 30;9:733688. doi: 10.3389/fcell.2021.733688 (PMC8514705; doi:10.3389/fcell.2021.733688)
Supplement: Supplementary file 1 [file Data_Sheet_1.docx]

Supplementary Material

**
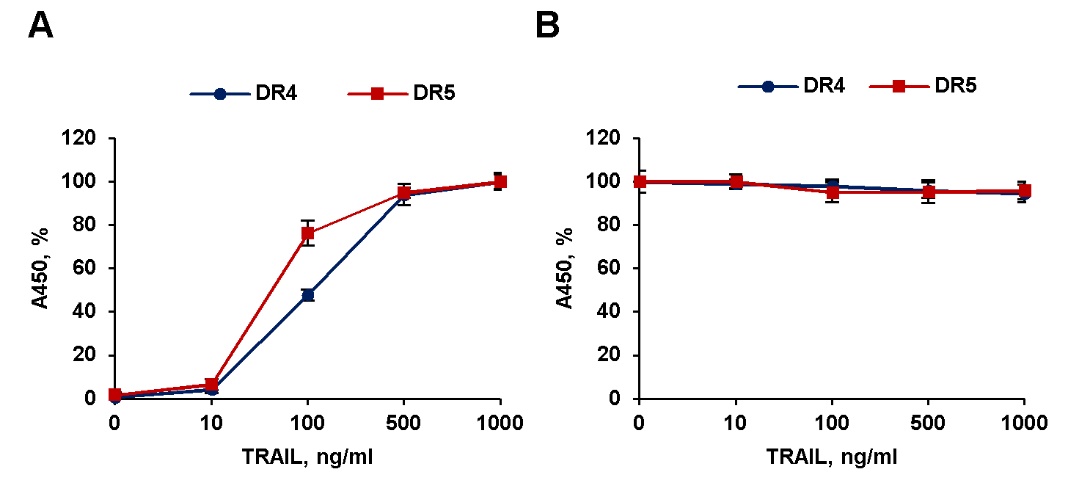
**

**Supplementary Figure 1.** TRAIL and anti-DR antibodies did not compete for binding to DR4 and DR5. **(A)** Binding of TRAIL to DRs. Recombinant extracellular domain of the DR5 and DR4 receptors (R&D Systems Inc., USA) were immobilized on ELISA plates overnight at 4°С at concentration of 1 μg/ml in sodium phosphate buffer (PBS). The plates were washed three times with PBST (PBS+ 0.05% Tween), and wells were blocked by 2% BSA in PBST for 1 h at room temperature. Then TRAIL was added to the wells at indicated concentrations and the plates were incubated for 1 h at 37°C. For detection of bounded TRAIL anti-TRAIL antibodies (MAB375, R&D systems, USA) was used. To evaluate the binding of TRAIL the mouse IgG (HAF007, R&D Systems, USA) conjugated with horseradish peroxidase was added to each well and the plates were incubated for 1 h and color was developed by OPD (o-phenylenediamine dihydrochloride) colorimetric substrate. After 15-minute incubation with substrate, reaction was stopped by 1 N H_2_SO_4_ solution. The optical density was determined at 450 nm by iMark spectrophotometer (Bio-Rad, USA). **(B)** The competitive binding of TRAIL and anti-DR antibodies to DR4 and DR5. The plate were captured with recombinant DRs as in (A) and the wells were incubated with TRAIL at concentration 1 μg/ml at 37°C for 1 h. After extensive washing of plates were incubated for 1 h at 37°C with anti-DR4 (DR-4-02) or anti-DR5 (DR5-01-1) monoclonal antibodies (GeneTex, Irvine, CA, USA) at concentration 5 μg/ml. The evaluation of antibody binding to the receptors was carried out as in (A). Data represent means ± SD of three independent experiments. Raw data are available in Table S1.


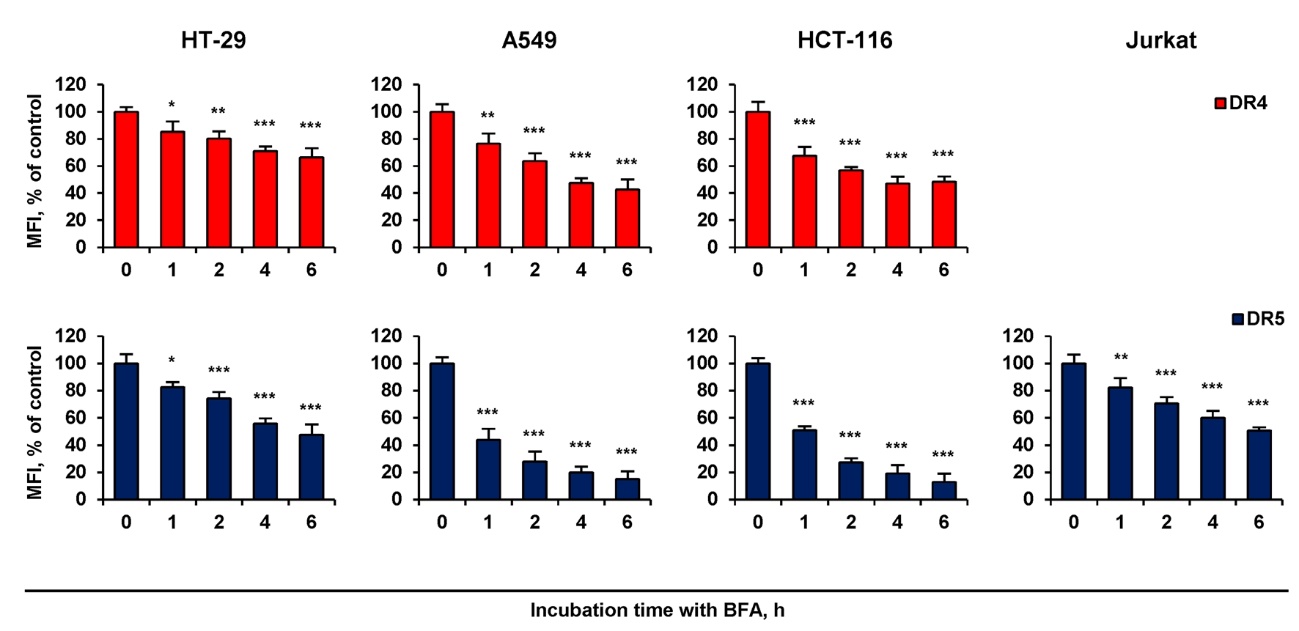


**Supplementary Figure 2.** Brefeldin A downregulated DR4 and DR5 surface expression in time-dependent manner. HT-29, A549, HCT116 and Jurkat cells were treated with 1.5 µM brefeldin A (BFA) at indicated periods and the surface expression of DR4 and DR5 was determined by flow cytometry. Mean Fluorescence Intensity (MFI) values are presented as a percentage relative to BFA non-treated cells. Data represent means ± SD of three independent experiments. * (p < 0.05), ** (p < 0.01) and *** (p < 0.001) indicated significant difference from the control according to One-way ANOVA followed by Dunnett’s posthoc test. Raw data are available in Table S2.

**
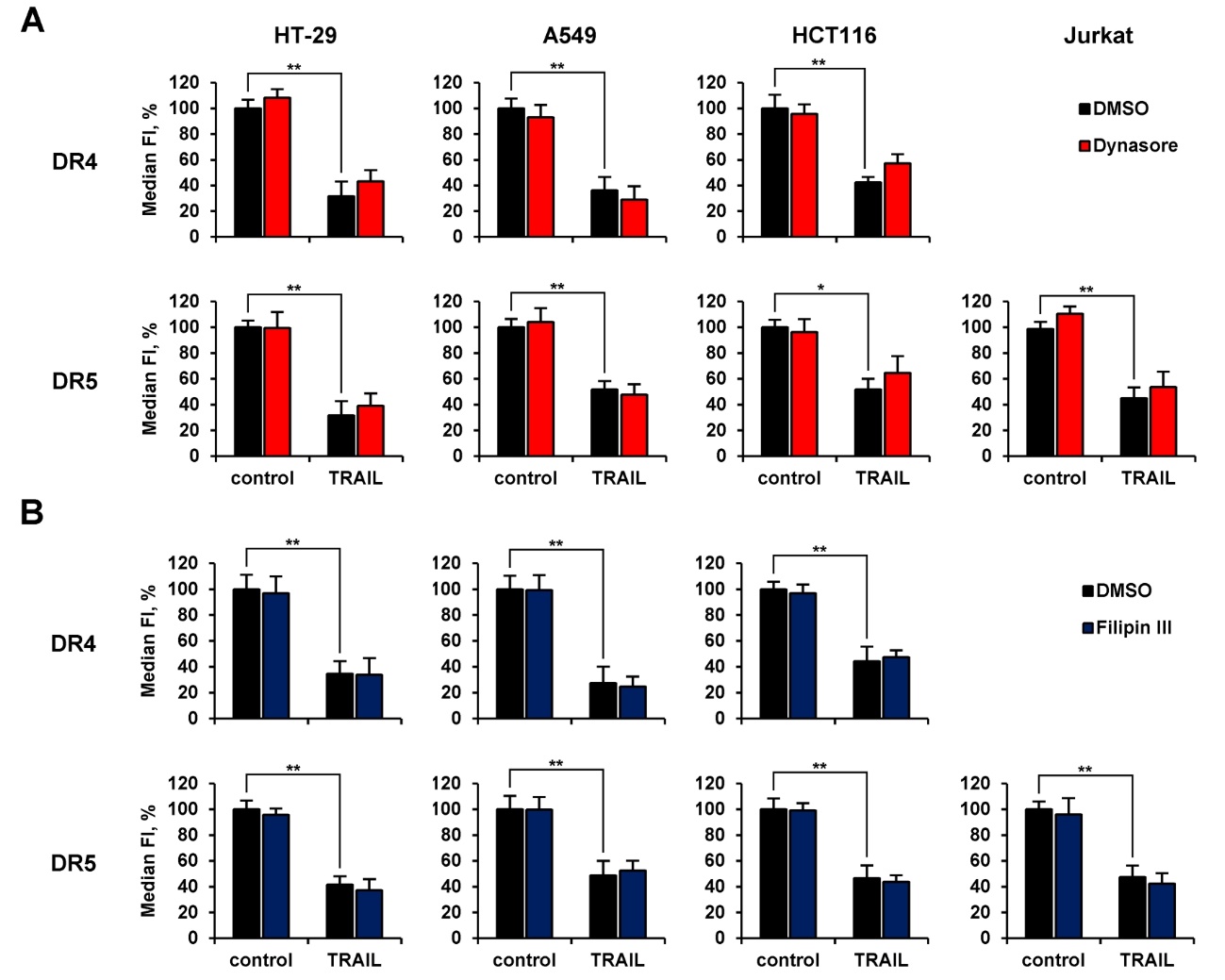
**

**Supplementary Figure 3.** Inhibitor of clathrin mediated endocytosis dynasore or cholesterol depleting agent filipin III did not significantly inhibit TRAIL mediated endocytosis of DR4 or DR5. HT-29, A549, HCT116 and Jurkat cells were treated with or without 80 µM dynasore **(A)** or 5 µM filipin III **(B)** for 1 h following 100 ng/ml TRAIL for another 1 h. The surface expression of DR4 and DR5 was determined by flow cytometry. Mean Fluorescence Intensity (MFI) values are presented as a percentage relative to non-treated with inhibitors cells. Data represent means ± SD of three independent experiments. * (p < 0.01) and ** (p < 0.001) indicate significant difference between groups according to One-way ANOVA followed by Tukey test. Raw data are available in Table S5.

**Supplementary Table 1. Raw data of enzyme-linked immunosorbent assay (ELISA) for Figure S1.**

| Receptors | TRAIL, ng/ml | **A_450_** | | | | | |
| --- | --- | --- | --- | --- | --- | --- | --- |
|  |  | Figure S1A | | | Figure S1B | | |
| DR4 | 0 | 0.012 | 0.007 | 0.008 | 2.000 | 1.953 | 1.937 |
|  | 10 | 0.050 | 0.091 | 0.042 | 1.989 | 1.923 | 1.917 |
|  | 100 | 0.629 | 0.692 | 0.689 | 1.986 | 1.908 | 1.876 |
|  | 500 | 1.254 | 1.301 | 1.390 | 1.877 | 1.957 | 1.819 |
|  | 1000 | 1.376 | 1.461 | 1.361 | 1.910 | 1.763 | 1.891 |
| DR5 | 0 | 0.021 | 0.036 | 0.009 | 1.240 | 1.190 | 1.315 |
|  | 10 | 0.096 | 0.045 | 0.099 | 1.276 | 1.198 | 1.266 |
|  | 100 | 0.894 | 0.888 | 1.014 | 1.127 | 1.193 | 1.240 |
|  | 500 | 1.131 | 1.188 | 1.152 | 1.122 | 1.196 | 1.255 |
|  | 1000 | 1.182 | 1.215 | 1.266 | 1.148 | 1.249 | 1.197 |

**Supplementary Table 2. Raw data of surface death receptors flow cytometry analysis for Figure S2.**

| Receptors | BFA, h | **Median FI** | | | | | | | | | | | |
| --- | --- | --- | --- | --- | --- | --- | --- | --- | --- | --- | --- | --- | --- |
|  |  | Cell lines | | | | | | | | | | | |
|  |  | HT-29 | | | A549 | | | HCT116 | | | Jurkat | | |
|  | isotype | 2961.2 | 2971.8 | 2981.2 | 2000.5 | 2083.0 | 2001.4 | 2214.8 | 2198.4 | 2208.4 | 966.6 | 942.1 | 1046.8 |
| DR5 | control | 10263.8 | 10221.4 | 11131.8 | 10145.3 | 10071.7 | 9464.6 | 15440.3 | 16018.9 | 16491.3 | 3024.2 | 3058.0 | 2913.6 |
|  | 1 | 9534.1 | 9159.4 | 8998.4 | 5275.4 | 5046.8 | 6171.2 | 8803.3 | 9409.6 | 9527.1 | 2478.5 | 2732.8 | 2724.9 |
|  | 2 | 9001.6 | 8332.9 | 8458.3 | 3532.9 | 4558.9 | 4556.6 | 5513.8 | 6300.4 | 6050.0 | 2261.2 | 2414.7 | 2479.7 |
|  | 4 | 7448.2 | 7219.4 | 6887.7 | 3495.9 | 3363.4 | 3919.4 | 3939.9 | 5600.0 | 4939.2 | 2247.7 | 2112.7 | 2136.0 |
|  | 6 | 7064.0 | 5934.7 | 6726.2 | 3200.9 | 2819.5 | 3648.9 | 3562.7 | 4983.1 | 3546.9 | 2078.6 | 1989.1 | 2061.1 |
| DR4 | control | 12295.8 | 11988.4 | 11715.5 | 21177.7 | 19292.0 | 20484.2 | 20506.8 | 22036.2 | 23365.3 | – | – | – |
|  | 1 | 11450.9 | 10246.5 | 10328.4 | 16267.5 | 14561.9 | 17161.3 | 14173.8 | 16772.7 | 15599.8 | – | – | – |
|  | 2 | 9889.6 | 9919.0 | 10678.0 | 12489.7 | 13849.0 | 14580.5 | 13252.1 | 14014.7 | 13150.4 | – | – | – |
|  | 4 | 9803.5 | 9180.9 | 9365.6 | 10657.4 | 10185.7 | 11364.8 | 12137.6 | 10388.7 | 11985.7 | – | – | – |
|  | 6 | 9670.2 | 8601.5 | 8679.1 | 9900.3 | 8545.5 | 11128.5 | 11971.8 | 10951.8 | 12385.3 | – | – | – |

–, not investigated

**Supplementary Table 3. Raw data of surface death receptors flow cytometry analysis for Figures 5B and 5C.**

| Receptors | Samples | | **Median FI** | | | | | | | | | | | |
| --- | --- | --- | --- | --- | --- | --- | --- | --- | --- | --- | --- | --- | --- | --- |
|  |  |  | Cell lines | | | | | | | | | | | |
|  |  |  | HT-29 | | | A549 | | | HCT116 | | | Jurkat | | |
| DR5 | BFA, μM,  6 h | isotype | 2856.8 | 2854.2 | 2956.3 | 2157.8 | 1968.5 | 2015.2 | 2420.2 | 2466.4 | 2301.3 | 1015.0 | 996.0 | 1026.9 |
|  |  | control | 12300.8 | 11403.8 | 12559.5 | 8817.7 | 9137.9 | 8954.5 | 16187.2 | 15105.5 | 16411.6 | 2339.3 | 2134.6 | 2251.0 |
|  |  | 0.1 | 7367.2 | 7736.6 | 8227.5 | 4857.5 | 4826.0 | 4031.6 | 14593.2 | 13604.7 | 14690.9 | 2315.5 | 2210.6 | 2258.3 |
|  |  | 0.5 | 6962.4 | 7091.0 | 7836.3 | 4460.8 | 4188.1 | 3876.2 | 6104.8 | 6683.6 | 5397.0 | 1457.3 | 1536.8 | 1489.1 |
|  |  | 1.0 | 6567.2 | 6759.0 | 7682.7 | 4201.1 | 3690.2 | 3511.7 | 5992.7 | 6364.7 | 4948.3 | 1472.6 | 1459.5 | 1378.8 |
|  | BFA, μM,  6 h after TRAIL washing | isotype | 2880.7 | 2878.4 | 3154.4 | 2221.0 | 2208.8 | 2277.7 | 2396.8 | 2362.2 | 2198.7 | 1086.7 | 1067.2 | 957.2 |
|  |  | control | 12645.8 | 14322.4 | 13799.0 | 10524.8 | 11373.8 | 11423.1 | 17785.2 | 18912.9 | 18776.2 | 2264.0 | 2286.0 | 2298.4 |
|  |  | TRAIL, 1 h | 3773.8 | 4438.8 | 4401.2 | 4316.2 | 4732.9 | 4594.3 | 6421.5 | 7219.5 | 6288.9 | 1402.6 | 1293.9 | 1232.7 |
|  |  | 0 | 15911.3 | 17216.9 | 16409.4 | 10904.6 | 11376.8 | 11088.6 | 16249.6 | 17471.1 | 16802.7 | 2423.6 | 2575.4 | 2467.2 |
|  |  | 0.1 | 5378.0 | 6098.8 | 5668.5 | 4190.1 | 4614.5 | 4177.5 | 14686.3 | 16995.5 | 16246.4 | 2174.5 | 2064.7 | 2032.4 |
|  |  | 0.5 | 3941.8 | 5438.7 | 5023.6 | 3591.6 | 3301.4 | 4155.6 | 7913.4 | 7953.1 | 9443.2 | 1833.3 | 1866.3 | 1804.9 |
|  |  | 1.0 | 3972.6 | 5072.8 | 4842.7 | 3405.1 | 3165.0 | 4127.2 | 8083.1 | 8425.0 | 9550.8 | 1942.8 | 1887.4 | 1880.3 |
| DR4 | BFA, μM,  6 h | isotype | 2914.9 | 3065.3 | 2896.1 | 2140.1 | 2091.2 | 2199.3 | 2624.6 | 2347.1 | 2242.2 | – | – | – |
|  |  | control | 9515.3 | 10141.3 | 10050.9 | 21866.7 | 23390.1 | 21242.3 | 28275.8 | 28828.3 | 26157.1 | – | – | – |
|  |  | 0.1 | 7861.7 | 8567.7 | 7982.1 | 16615.6 | 16714.5 | 15656.6 | 23496.5 | 24462.4 | 23305.4 | – | – | – |
|  |  | 0.5 | 7897.3 | 8393.3 | 7971.7 | 13379.1 | 16289.2 | 13972.9 | 20028.4 | 20926.2 | 19998.5 | – | – | – |
|  |  | 1.0 | 7824.9 | 7828.1 | 7369.7 | 16396.7 | 16316.7 | 14782.6 | 19099.8 | 21424.1 | 19199.3 | – | – | – |
|  | BFA, μM,  6 h after TRAIL washing | isotype | 3017.5 | 3113.1 | 2846.7 | 2005.2 | 2122.6 | 2249.4 | 2253.8 | 2056.5 | 2227.3 | – | – | – |
|  |  | control | 10533.8 | 11190.6 | 11109.7 | 22185.7 | 21974.8 | 24683.1 | 29327.6 | 26307.1 | 26434.7 | – | – | – |
|  |  | TRAIL, 1 h | 3824.2 | 3829.1 | 4046.0 | 4751.4 | 5326.1 | 4273.6 | 5128.1 | 6147.4 | 4668.0 | – | – | – |
|  |  | 0 | 10526.8 | 10771.7 | 11069.2 | 25267.5 | 24680.0 | 24584.8 | 22037.4 | 24327.9 | 23662.0 | – | – | – |
|  |  | 0.1 | 4043.6 | 4580.2 | 4129.0 | 15425.1 | 15251.7 | 18164.1 | 14713.9 | 16904.1 | 15001.0 | – | – | – |
|  |  | 0.5 | 3569.9 | 4543.6 | 3822.9 | 15132.3 | 13988.1 | 17033.0 | 9346.9 | 11100.9 | 9913.9 | – | – | – |
|  |  | 1.0 | 3690.2 | 4261.0 | 3704.6 | 15562.7 | 14327.1 | 16373.6 | 10154.2 | 11547.3 | 9181.7 | – | – | – |

–, not investigated

**Supplementary Table 4. Raw data of surface death receptors flow cytometry analysis for Figures 6A and 6D.**

| Receptors | Samples | | **Median FI** | | | | | | | | | | | |
| --- | --- | --- | --- | --- | --- | --- | --- | --- | --- | --- | --- | --- | --- | --- |
|  |  |  | Cell lines | | | | | | | | | | | |
|  |  |  | HT-29 | | | A549 | | | HCT116 | | | Jurkat | | |
| DR5 | CHX, μg/ml,  6 h | isotype | 2942.3 | 3155.1 | 2990.9 | 2283.2 | 2300.1 | 2221.8 | 2292.7 | 2520.6 | 2260.5 | 1016.7 | 1027.2 | 927.2 |
|  |  | control | 12420.2 | 13842.7 | 12805.8 | 8566.7 | 9107.2 | 8512.8 | 16516.9 | 17235.1 | 18103.8 | 2381.3 | 2466.8 | 2388.7 |
|  |  | 2 | 12381.3 | 13552.8 | 13848.2 | 7721.1 | 8395.7 | 8316.8 | 12699.1 | 14866.2 | 14488.4 | 2334.1 | 2434.1 | 2344.1 |
|  |  | 20 | 12428.6 | 13232.8 | 12532.3 | 6119.6 | 6411.5 | 5413.7 | 9117.3 | 9936.1 | 9549.2 | 2232.2 | 2245.5 | 2197.8 |
|  |  | 50 | 11312.3 | 11754.7 | 11856.5 | 5618.0 | 6071.2 | 5176.0 | 8546.4 | 9973.9 | 9588.3 | 2218.1 | 2285.3 | 2278.4 |
|  | CHX, μg/ml,  6 h after TRAIL washing | isotype | 2802.3 | 3034.4 | 3067.9 | 2248.6 | 2273.6 | 2236.9 | 2085.3 | 1916.2 | 1931.6 | 947.5 | 996.7 | 939.5 |
|  |  | control | 10396.2 | 10391.6 | 11068.1 | 9265.7 | 10229.3 | 10207.1 | 15618.8 | 15058.8 | 16507.7 | 2824.5 | 3034.6 | 2970.4 |
|  |  | TRAIL, 1 h | 3846.0 | 3769.9 | 4270.8 | 4221.8 | 4294.5 | 3839.8 | 6066.0 | 6619.4 | 5833.9 | 1500.2 | 1602.9 | 1584.8 |
|  |  | 0 | 12024.6 | 12109.4 | 13233.5 | 9988.2 | 10152.4 | 10306.5 | 14296.1 | 13847.4 | 15819.5 | 2930.7 | 3241.3 | 3039.0 |
|  |  | 2 | 10281.9 | 10000.2 | 10975.3 | 6688.7 | 6326.7 | 7197.3 | 9867.8 | 10303.9 | 10487.2 | 2317.2 | 2287.8 | 2501.2 |
|  |  | 20 | 7852.1 | 7883.9 | 8659.8 | 4903.4 | 4468.5 | 4644.7 | 5708.4 | 6606.8 | 7561.2 | 2007.3 | 2128.3 | 2215.5 |
|  |  | 50 | 8031.4 | 7412.8 | 8323.8 | 3918.8 | 3960.5 | 4678.0 | 7216.6 | 6226.0 | 7226.5 | 2161.6 | 2385.2 | 2143.3 |
| DR4 | CHX, μg/ml,  6 h | isotype | 3149.0 | 3120.6 | 3048.9 | 2103.3 | 2008.1 | 2078.0 | 2305.9 | 2222.5 | 2437.1 | – | – | – |
|  |  | control | 11474.9 | 10553.3 | 11127.4 | 21873.2 | 22103.5 | 24140.2 | 26201.8 | 25716.4 | 27635.3 | – | – | – |
|  |  | 2 | 9236.5 | 8970.8 | 9969.4 | 21311.1 | 22232.1 | 22747.2 | 25917.4 | 27275.0 | 27560.4 | – | – | – |
|  |  | 20 | 8466.2 | 8958.6 | 8774.5 | 21428.7 | 22228.1 | 22853.5 | 26154.4 | 27508.5 | 27722.1 | – | – | – |
|  |  | 50 | 8767.5 | 9344.7 | 9421.3 | 20349.8 | 21076.7 | 21328.2 | 27418.8 | 28246.3 | 24970.9 | – | – | – |
|  | CHX, μg/ml,  6 h after TRAIL washing | isotype | 3014.2 | 2889.4 | 2853.3 | 2212.6 | 2228.1 | 2251.8 | 1960.7 | 2141.1 | 2124.2 | – | – | – |
|  |  | control | 10255.2 | 10774.3 | 10762.6 | 22995.7 | 25589.1 | 25610.8 | 20331.6 | 22763.2 | 22744.4 | – | – | – |
|  |  | TRAIL, 1 h | 3410.0 | 3672.0 | 3963.1 | 5371.3 | 5434.7 | 6669.6 | 5497.3 | 4989.1 | 4596.6 | – | – | – |
|  |  | 0 | 9383.0 | 10175.2 | 10342.6 | 24806.7 | 25727.5 | 25906.1 | 18724.3 | 18270.1 | 16730.3 | – | – | – |
|  |  | 2 | 5704.8 | 5098.9 | 5010.1 | 23553.4 | 22767.5 | 25110.5 | 13592.0 | 11582.1 | 10745.1 | – | – | – |
|  |  | 20 | 4729.6 | 4496.2 | 5152.3 | 23967.7 | 23236.0 | 24760.5 | 9341.5 | 8553.9 | 7875.2 | – | – | – |
|  |  | 50 | 4114.6 | 3870.3 | 4431.0 | 21328.0 | 23466.2 | 23717.5 | 9949.7 | 8178.1 | 7618.3 | – | – | – |

–, not investigated

**Supplementary Table 5. Raw data of surface death receptors flow cytometry analysis for Figure S3.**

| Figures | Receptors | Samples | **Median FI** | | | | | | | | | | | |
| --- | --- | --- | --- | --- | --- | --- | --- | --- | --- | --- | --- | --- | --- | --- |
|  |  |  | Cell lines | | | | | | | | | | | |
|  |  |  | HT-29 | | | A549 | | | HCT116 | | | Jurkat | | |
| S3A |  | isotype | 3084.9 | 3166.3 | 3175.9 | 2286.4 | 2242.3 | 2242.5 | 2287.3 | 2235.5 | 2246.1 | 1210.9 | 959.5 | 909.8 |
|  | DR5 | control | 13530.6 | 12776.8 | 12687.9 | 11071.9 | 10976.4 | 10058.6 | 14519.5 | 13783.0 | 15210.2 | 2879.0 | 2776.6 | 2756.3 |
|  |  | TRAIL | 4960.8 | 6828.1 | 7051.0 | 6718.3 | 7106.8 | 6007.7 | 8874.0 | 7454.3 | 9430.2 | 1863.8 | 1780.1 | 1853.1 |
|  |  | Dynasore | 11538.0 | 13798.9 | 13601.1 | 10158.2 | 11144.8 | 11911.8 | 14592.5 | 12590.5 | 14919.0 | 3010.2 | 2934.0 | 2887.7 |
|  |  | Dynasore + TRAIL | 5879.8 | 7490.5 | 7700.1 | 6622.5 | 6772.3 | 5510.2 | 10437.5 | 8424.6 | 11579.2 | 1920.4 | 2030.2 | 1993.4 |
|  | DR4 | control | 10999.2 | 10999.0 | 12001.6 | 19794.1 | 20506.4 | 17923.8 | 25280.1 | 22189.8 | 26925.7 | – | – | – |
|  |  | TRAIL | 5539.1 | 4882.1 | 6746.6 | 8920.9 | 9985.9 | 6563.7 | 12117.8 | 10736.6 | 12541.0 | – | – | – |
|  |  | Dynasore | 12252.8 | 11413.0 | 12358.9 | 19501.1 | 18866.6 | 16289.7 | 25489.2 | 22060.1 | 24007.8 | – | – | – |
|  |  | Dynasore + TRAIL | 6891.3 | 5901.0 | 7286.1 | 7298.4 | 8992.1 | 5419.9 | 15475.1 | 13397.3 | 16566.2 | – | – | – |
| S3B |  | isotype | 3257.8 | 3169.1 | 3173.0 | 2274.2 | 2214.1 | 2383.6 | 2368.9 | 2481.4 | 2824.1 | 957.0 | 943.4 | 1003.8 |
|  | DR5 | control | 12945.5 | 14279.8 | 13553.6 | 7935.3 | 7081.2 | 7034.7 | 14544.4 | 13245.5 | 13185.8 | 3119.0 | 3007.9 | 2916.8 |
|  |  | TRAIL | 7025.4 | 8253.5 | 7238.9 | 4512.1 | 5220.8 | 4262.7 | 7184.7 | 8909.8 | 7148.4 | 2069.9 | 1990.4 | 1773.1 |
|  |  | Filipin III | 12964.5 | 13731.9 | 12784.2 | 7889.0 | 6908.6 | 7261.7 | 14113.0 | 13253.8 | 13377.8 | 2991.4 | 3122.4 | 2680.1 |
|  |  | Filipin III + TRAIL | 7048.2 | 7995.4 | 6234.7 | 4844.9 | 5301.4 | 4723.1 | 7430.4 | 7808.9 | 7017.5 | 1953.3 | 1868.6 | 1682.0 |
|  | DR4 | control | 9964.4 | 11178.7 | 9751.6 | 27677.6 | 28697.3 | 24038.7 | 25171.7 | 22809.5 | 24012.6 | – | – | – |
|  |  | TRAIL | 5299.2 | 6436.5 | 5294.5 | 11674.9 | 9713.2 | 5651.1 | 11865.9 | 9555.7 | 14822.0 | – | – | – |
|  |  | Filipin III | 9294.5 | 11022.4 | 9914.2 | 28765.1 | 27695.7 | 23541.3 | 24283.8 | 21610.0 | 23983.5 | – | – | – |
|  |  | Filipin III + TRAIL | 4739.4 | 6479.0 | 5580.1 | 9698.4 | 9169.0 | 6302.6 | 13292.9 | 11279.2 | 13519.6 | – | – | – |

–, not investigated

**Supplementary Table 6. Raw data of surface death receptors flow cytometry analysis for Figures 7A and 7B.**

| Receptors | Samples | **Median FI** | | | | | | | | | | | |
| --- | --- | --- | --- | --- | --- | --- | --- | --- | --- | --- | --- | --- | --- |
|  |  | Cell lines | | | | | | | | | | | |
|  |  | HT-29 | | | A549 | | | HCT116 | | | Jurkat | | |
|  | isotype | 2962.3 | 3074.6 | 2937.1 | 2075.4 | 2074.8 | 2102.8 | 2168.3 | 2059.7 | 2043.5 | 1171.3 | 1096.6 | 1175.7 |
| DR5 | control | 12396.1 | 13067.9 | 13488.9 | 10802.7 | 10803.6 | 10327.6 | 15314.0 | 16663.0 | 15923.9 | 3386.7 | 3006.1 | 3148.3 |
|  | TRAIL | 6385.7 | 5024.7 | 6053.2 | 4616.7 | 4977.6 | 5658.2 | 8581.3 | 8036.9 | 9711.7 | 2137.6 | 2261.9 | 2187.6 |
|  | sucrose | 13830.0 | 13681.4 | 13961.3 | 10141.7 | 10771.2 | 11421.8 | 17080.1 | 16527.6 | 17284.0 | 3564.7 | 3647.8 | 3518.8 |
|  | sucrose + TRAIL | 8390.5 | 10261.4 | 8943.5 | 8729.6 | 8527.3 | 9528.3 | 12192.7 | 11215.4 | 12714.9 | 2462.5 | 2619.8 | 2513.3 |
| DR4 | control | 10296.4 | 11131.5 | 11549.7 | 21191.7 | 20910.1 | 22594.5 | 24586.7 | 26113.2 | 26099.0 | – | – | – |
|  | TRAIL | 5299.0 | 5850.9 | 6124.5 | 8607.4 | 9095.1 | 10033.5 | 13358.2 | 15542.6 | 13457.8 | – | – | – |
|  | sucrose | 11415.7 | 11835.5 | 10800.1 | 20361.3 | 20962.4 | 21566.4 | 32512.2 | 31595.0 | 30486.5 | – | – | – |
|  | sucrose + TRAIL | 8536.7 | 9485.1 | 8764.3 | 15191.0 | 15226.5 | 16423.1 | 25305.8 | 22378.6 | 25787.8 | – | – | – |

–, not investigated

**Supplementary Table 7. Raw data of WST-1 assay for Figure 7C.**

| Samples | **A_450_ - A_655_** | | | | | | | | | | | |
| --- | --- | --- | --- | --- | --- | --- | --- | --- | --- | --- | --- | --- |
|  | Cell lines | | | | | | | | | | | |
|  | HT-29 | | | A549 | | | HCT116 | | | Jurkat | | |
| control | 1.470 | 1.457 | 1.452 | 1.792 | 1.748 | 1.713 | 1.046 | 1.093 | 1.118 | 1.451 | 1.550 | 1.596 |
| sucrose | 1.003 | 0.987 | 1.061 | 1.414 | 1.420 | 1.455 | 0.876 | 0.913 | 0.871 | 1.237 | 1.220 | 1.226 |
| sucrose + Z-VAD-FMK | 0.983 | 1.078 | 0.867 | 1.282 | 1.365 | 1.310 | 0.933 | 0.928 | 0.941 | 1.139 | 1.146 | 1.192 |
| TRAIL | 1.490 | 1.444 | 1.431 | 1.665 | 1.572 | 1.695 | 0.683 | 0.697 | 0.710 | 1.521 | 1.495 | 1.486 |
| sucrose + TRAIL | 0.462 | 0.405 | 0.356 | 0.724 | 0.766 | 0.634 | 0.463 | 0.464 | 0.461 | 0.885 | 0.868 | 0.816 |
| sucrose + Z-VAD-FMK + TRAIL | 1.018 | 0.993 | 1.085 | 1.163 | 1.157 | 1.148 | 0.943 | 0.934 | 0.903 | 1.156 | 0.985 | 1.017 |
